# Dopamine D1 Receptor (D1R) Expression Is Controlled by a Transcriptional Repressor Complex Containing DISC1

**DOI:** 10.1007/s12035-019-1566-6

**Published:** 2019-03-26

**Authors:** Yeongjun Suh, Su-Jin Noh, Saebom Lee, Bo Kyoung Suh, Su Been Lee, Jinhyuk Choi, Jaehoon Jeong, Sangjune Kim, Sang Ki Park

**Affiliations:** 1 Department of Life Sciences, Pohang University of Science and Technology, Pohang, 37673 Republic of Korea; 2 The Russell H. Morgan Department of Radiology and Radiological Sciences, The Johns Hopkins University of School of Medicine, Baltimore, MD USA; 3 The Center for Nanomedicine at Wilmer Eye Institute, The Johns Hopkins University of School of Medicine, Baltimore, MD USA; 4 National Institute of Neurological Disorders and Stroke, National Institutes of Health, Bethesda, MD USA; 5 Neurodegeneration and Stem Cell Programs, Institute for Cell Engineering, The Johns Hopkins University of School of Medicine, Baltimore, MD USA; 6 Department of Neurology, The Johns Hopkins University School of Medicine, Baltimore, MD USA

**Keywords:** Dopamine D1 receptor, DISC1, KLF16, Transcriptional repression, SIN3A

## Abstract

Disrupted-in-Schizophrenia 1 (DISC1) is a scaffold protein implicated in various psychiatric diseases. Dysregulation of the dopamine system has been associated with DISC1 deficiency, while the molecular mechanism is unclear. In this study, we propose a novel molecular mechanism underlying the transcriptional regulation of the dopamine D1 receptor (D1R) in the striatum via DISC1. We verified the increase in D1R at the transcriptional level in the striatum of DISC1-deficient mouse models and altered histone acetylation status at the *D1r* locus. We identified a functional interaction between DISC1 and Krüppel-like factor 16 (KLF16). KLF16 translocates DISC1 into the nucleus and forms a regulatory complex by recruiting SIN3A corepressor complexes to the *D1r* locus. Moreover, DISC1-deficient mice have altered D1R-mediated signaling in the striatum and exhibit hyperlocomotion in response to cocaine; the blockade of D1R suppresses these effects. Taken together, our results suggest that nuclear DISC1 plays a critical role in the transcriptional regulation of D1R in the striatal neuron, providing a mechanistic link between DISC1 and dopamine-related psychiatric symptoms.

**Electronic supplementary material:**

The online version of this article (10.1007/s12035-019-1566-6) contains supplementary material, which is available to authorized users.

## Introduction

Disruptions in dopamine signaling are critical factors in the pathophysiology of a number of mental disorders, such as schizophrenia and addictive disorders [1, 2]. In the dorsal and ventral striatum, dopamine D1 receptor (D1R), a primary D1-like dopamine receptor, plays crucial roles in motor control and reward process [3, 4]. Multiple psychiatric conditions are attributable, at least in part, to imbalances in dopaminergic pathways that signal D1R and dopamine D2 receptor (D2R) in the striatum. For example, in human subjects, the dysregulation of ligand binding to striatal dopamine receptors is associated with drug abuse and schizophrenia [5]. Furthermore, an altered ratio of D1R to D2R in the mouse striatum is connected to long-term drug responses [6].

The *Disc1* gene was initially discovered as a susceptibility factor for schizophrenia and related psychiatric conditions [7]. Subsequent studies have revealed crucial roles of disrupted-in-Schizophrenia 1 (DISC1) in neurodevelopment and neural processes, including neurogenesis, neuronal migration, synapse formation, and neurotransmission [8, 9]. Lines of evidence indicate that DISC1 dysfunction is associated with abnormalities of the dopamine system, such as lower dopamine level, upregulated mRNA levels of dopamine receptors, and hyperlocomotive activity after amphetamine administration in animal models [10]. Transgenic mouse models expressing dominant-negative DISC1 showed an altered dopaminergic transmission and elevated mRNA levels of dopamine receptors in the striatum as well as abnormal dopamine-related behaviors in response to methamphetamine administration [11]. Similarly, a DISC1 haploinsufficiency model expresses upregulated mRNA levels of dopamine receptors in the NAc [12]. However, the molecular mechanism underlying the effects of DISC1 remains to be elucidated.

KLF16 belongs to the Sp/Krüppel-like transcription factor family and binds to GC and GT boxes in the D1A and D2 dopamine receptor promoters with Cys-2-His-2 zinc finger motifs [13]. KLF16 interacts with SIN3A and recruits HDACs to function as a transcription regulator [14]. Thus, we hypothesized that DISC1 participates in the transcriptional regulation of dopamine receptors via KLF16, thereby influencing dopamine signaling.

In this study, we discovered a functional interaction between DISC1 and KLF16. This interaction induces the translocation of DISC1 into the nucleus and forms a regulatory complex with the SIN3A corepressor at the *D1r* locus in the mouse striatum. We show that the DISC1-deficient mouse model has an increased level of D1R in the striatum and exaggerated cocaine-induced hyperlocomotion, which is suppressed by the blockade of D1R. Our findings provide a novel route for the epigenetic regulation of dopamine signaling.

## Results

### DISC1 Affects the Transcription of *D1r* in the Mouse Striatum

**Fig. 1:**
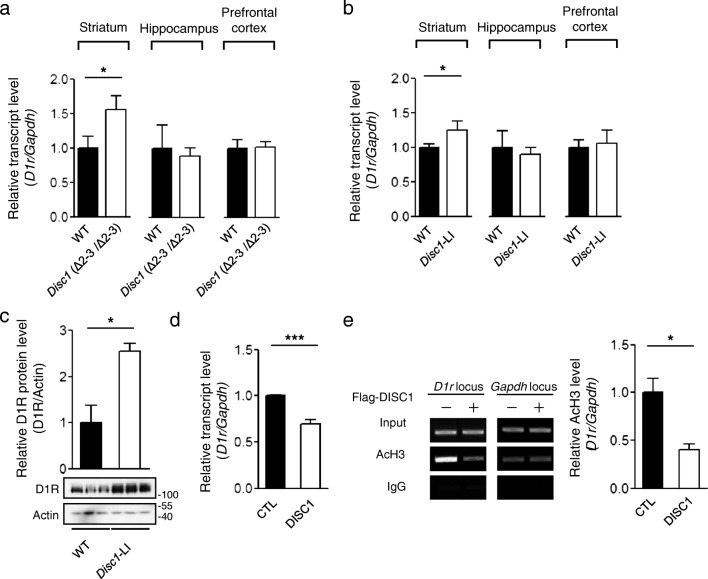
Upregulated *D1r* transcription in DISC1-deficient mice. **a** Relative transcript abundance of *D1r* in the striatum (*n* = 12), hippocampus (*n* = 6), and prefrontal cortex (*n* = 6) of adult (12-week-old) *Disc1* (Δ2–3/Δ2–3) mice (right) and those of wild-type (WT) B6 mice (left) assayed by real-time qRT-PCR. **b** Relative transcript abundance of *D1r* in the striatum (*n* = 6), hippocampus (*n* = 10), and prefrontal cortex (*n* = 6) of *Disc1-*LI mice (right), compared with those of WT B6 mice (left). **c** Increased protein level of D1R in the striatum of adult *Disc1-*LI mice (*n* = 3). **d** Reduced transcript level of *D1r* in differentiated CAD cells upon overexpression of Flag-DISC1 (*n* = 8). **e** Decreased acetylation level of histone H3 in differentiated CAD cells upon Flag-DISC1 overexpression (*n* = 3). Anti-AcH3 (K9 and K14) antibody was used for IP. Columns represent mean ± SEM. *P < 0.05; ***P < 0.001; two-tailed *t* test

We performed microarray analyses of the transcriptional profiles of primary neurons cultured from DISC1-deficient mouse embryos and observed that *D1r* mRNA showed significant upregulation compared with wild-type. To further confirm this preliminary observation, we used two DISC1-mutant mouse models: *Disc1* (Δ2–3/Δ2–3) mice and *Disc1*-LI mice [15, 16]. To quantify the *D1r* mRNA expression, we performed real-time quantitative reverse transcription PCR (qRT-PCR) using the dissected medial prefrontal cortex, striatum, and hippocampus of adult male mouse brains, which are the primary brain subdomains for dopamine receptor expression. The *D1r* mRNA level was significantly higher in the striatal region of *Disc1* (Δ2–3/Δ2–3) mice than in wild-type mice, while no significant differences were detected in other regions (Fig. 1a). The elevation of *D1r* mRNA in the striatum was consistent in *Disc1*-LI mice (Fig. 1b). We confirmed that the upregulated transcript abundance of *D1r* led to an increase in endogenous protein level in the striatum in *Disc1*-LI mice (Fig. 1c).

To examine if DISC1 affects other types of dopamine receptors, we performed qRT-PCR using the striatal tissue of adult *Disc1*-LI mice. Among the five subtypes of dopamine receptors, only D1R showed an alteration in the transcript level in the striatum of *Disc1*-LI mice (Supplementary Fig. 1). Compared with D1R and D2R, the transcript abundances of dopamine D3 receptor (D3R) and dopamine D4 receptor (D4R) were barely detectable in the striatum, and dopamine D5 receptor (D5R) transcript was not detectable under the same experimental condition, which is consistent with previous study [17]. These results suggest that the involvement of DISC1 in transcription in the striatum is relatively specific for D1R.

We also evaluated its effect on *D1r* mRNA in differentiated Cath.a-differentiated (CAD) cells with considerable dopamine receptor expression [18]. The overexpression of Flag-tagged mouse DISC1 in differentiated CAD cells induced the upregulation of *D1r* mRNA (Fig. 1d), opposite to the observations in the DISC1-deficient mice. To determine if the transcriptional upregulation is associated with the chromatin status of the *D1r* locus, we employed chromatin immunoprecipitation (ChIP) assay using an anti-acetylated histone H3 (AcH3) antibody. The level of AcH3 at the *D1r* locus was remarkably lower in the DISC1-overexpressing CAD cells than in control cells (Fig. 1e). Collectively, these results indicate that DISC1 affects *D1r* transcription via the alteration of the chromatin acetylation status at the *D1r* locus.

### KLF16 Translocates DISC1 into the Nucleus and Forms a Corepressor Complex at the *D1r* Locus

**Fig. 2:**
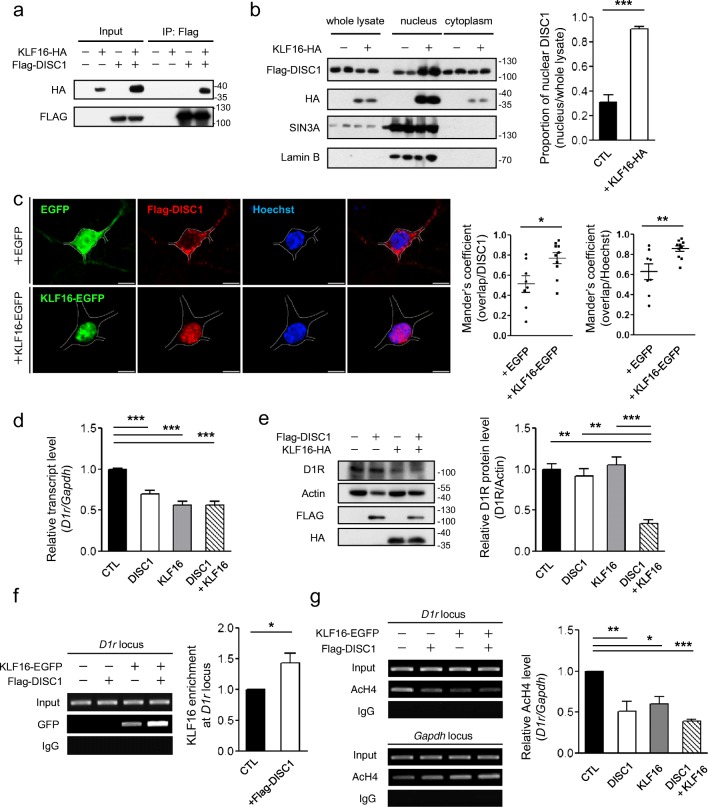
Translocation of DISC1 into the nucleus via interaction with KLF16. **a** CoIP of KLF16-HA with Flag-DISC1 using anti-Flag antibody from differentiated CAD cell lysates. **b** Increased DISC1 protein level in the nucleus-enriched fractions of differentiated CAD cell lysates (*n* = 3). **c** Confocal images of Flag-DISC1 (red) and the Hoechst 33342 (blue) upon EGFP or KLF16-EGFP (green) overexpression. Mander’s overlap coefficients between Flag-DISC1 and Hoechst were calculated by the Cellsens software (Olympus) (*n* = 8 for EGFP, *n* = 10 for KLF16-EGFP, E15.5 mouse striatal cultured neurons at DIV10). The white line outlines the neuronal soma. The scale bar represents 5 μm. **d** Reduced transcript level of *D1r* upon DISC1, KLF16, and their combined overexpression in differentiated CAD cells (n = 8). **e** Reduced endogenous D1R protein level upon DISC1 and KLF16 co-expression (*n* = 3). **f** Enhanced ChIP-PCR signal of KLF16-EGFP at the *D1r* locus in DISC1-cotransfected CAD cells (*n* = 5). Anti-GFP antibody was used for IP. **g** Reduced acetylated H4 level at the *D1r* locus upon DISC1, KLF16, and their combined overexpression in differentiated CAD cells (*n* = 4). Anti-AcH4 (K5, K8, K12, and K16) antibody was used for IP. Data represent mean ± SEM. *P < 0.05; **P < 0.01; ***P < 0.001; two-tailed unpaired *t* test (**b, c**), paired *t* test (**f**); one-way ANOVA with post-hoc Tukey test (**d**, **e, g**)

Potential roles of DISC1 in the nucleus have been suggested [19], but a clear transcriptional mechanism by which DISC1 regulates target genes has not been elucidated. Given that the expression of dopamine receptors is regulated by Krüppel-like factor family transcription factors [13, 20], we evaluated if KLF16 interacted with DISC1 by co-immunoprecipitation (coIP) using differentiated CAD cells and observed a significant coIP signal (Fig. 2a). Moreover, in a nuclear fractionation experiment, we found that the DISC1 protein, which mainly resides in the cytosol [21], was more highly expressed in the nuclear fraction when KLF16 was co-expressed (Fig. 2b). This result was further supported by immunocytochemistry in primary cultured mouse striatal neurons at DIV10 (Fig. 2c). KLF16 is a nuclear shuttling protein; accordingly, these results indicate that KLF16 interacts with DISC1 and they are co-translocated into the nucleus.

To verify whether DISC1 and KLF16 interact to regulate *D1r* transcription, we first examined the expression level of *D1r* by real-time qRT-PCR and western blot assay in differentiated CAD cells upon DISC1 and KLF16 co-expression. DISC1 and KLF16 overexpression significantly reduced the level of *D1r* transcription (Fig. 2d), which led to a decrease in endogenous D1R protein level (Fig. 2e). Moreover, in the ChIP assay, we found that KLF16 was associated with the *D1r* locus when it was co-expressed with DISC1 (Fig. 2f). Acetylated histone H4 signal at the *D1r* locus was reduced by DISC1, KLF16, and their combined expression (Fig. 2g). Taken together, these results suggest that DISC1 and KLF16 form a protein complex to negatively regulate *D1r* transcription, which is achieved by the alteration of chromatin acetylation at the *D1r* locus.

### DISC1 and KLF16 Recruit the SIN3A Repressor Complex to the *D1r* Locus

**Fig. 3:**
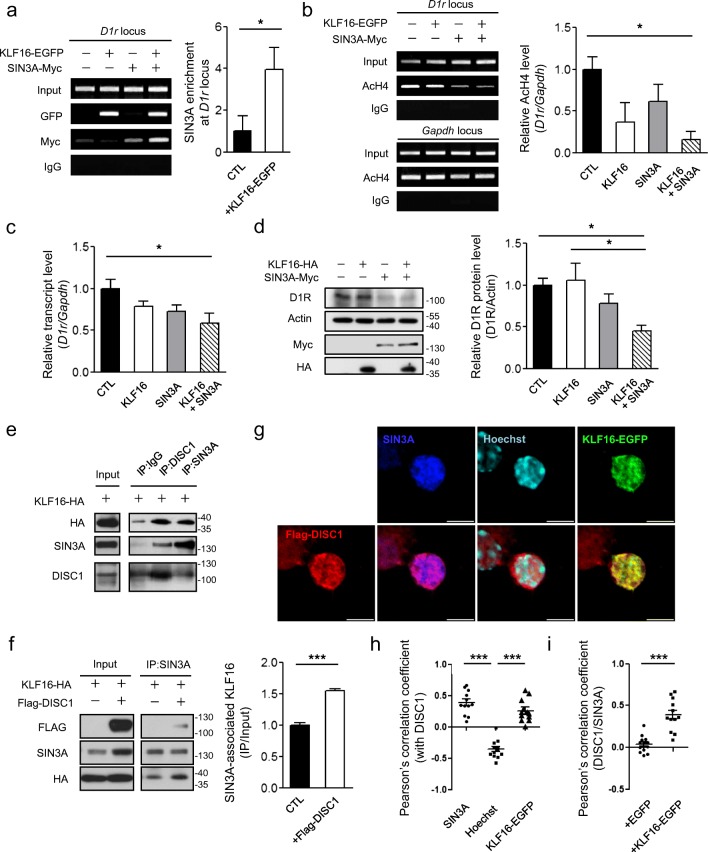
Recruitment of SIN3A corepressor by the KLF16-DISC1 complex at the *D1r* locus. **a** Enhanced ChIP-PCR signal of SIN3A-Myc at the *D1r* locus upon co-expression of KLF16 in differentiated CAD cells (*n* = 3). Antibody against GFP and Myc were used for IP. **b** Reduced acetylated H4 level at the *D1r* locus upon KLF16 and SIN3A overexpression in differentiated CAD cells (*n* = 3). Anti-AcH4 (K5, K8, K12, and K16) antibody was used for IP. **c** Reduced transcript abundance of *D1r* upon KLF16 and SIN3A overexpression in differentiated CAD cells (*n* = 6). **d** Reduced endogenous D1R protein level upon KLF16 and SIN3A overexpression in differentiated CAD cells (*n* = 4). **e** CoIP of KLF16-HA with endogenous DISC1 and SIN3A from differentiated CAD cell lysate. **f** Enhanced coIP of KLF16-HA with endogenous SIN3A upon Flag-DISC1 overexpression in differentiated CAD cells (n = 3). Anti-SIN3A antibody was used for IP. **g** Confocal images of Flag-DISC1 (red, left bottom), SIN3A (blue), Hoechst (cyan), KLF16-EGFP (green), and their merged images (bottom) of the nucleus of cultured mouse striatal neuron at DIV10. Scale bars represent 5 μm. **h** Pearson’s correlation coefficients between DISC1 and SIN3A, between DISC1 and Hoechst, and between DISC1 and KLF16-EGFP (*n* = 12). **i** Effect of KLF16-EGFP overexpression on the Pearson’s correlation coefficient between Flag-DISC1 and SIN3A (*n* = 14 for EGFP; *n* = 12 for KLF16-EGFP). Confocal images were obtained from at least three separate coverslips. Deconvoluted images and Pearson’s correlation coefficients were obtained by the CellSens software (Olympus). *P < 0.05; ***P < 0.001; one-way ANOVA with post-hoc Tukey test (**b**, **c**, **d**, **h**); two-tailed *t* test (**a**, **f**, **i**)

KLF16 regulates the transcription of a target gene by recruiting the corepressor complex to the target locus [14]. SIN3A is a component of a corepressor complex associated with histone deacetylases [22]. Thus, we hypothesized that KLF16 directly binds to the *D1r* promoter region, thereby recruiting the SIN3A-containing repressor complex. To test this hypothesis, we performed a ChIP assay. The ChIP signal of SIN3A was enhanced by KLF16 co-expression at the *D1r* locus, while the accumulation of KLF16 at the *D1r* locus was not affected by SIN3A overexpression (Fig. 3a). Also, the acetylated histone H4 level at the *D1r* locus was decreased upon KLF16 and SIN3A co-expression (Fig. 3b) and it was further confirmed by qRT-PCR and western blot assay under KLF16 and SIN3A co-expression conditions (Fig. 3c and d). Collectively, these results indicate that KLF16 regulates *D1r* transcription by recruiting the SIN3A corepressor complex to the *D1r* promoter region and mediates changes in the chromatin state by histone deacetylation.

To further clarify the role of DISC1 in this process, we tested the physical interaction between endogenous SIN3A and DISC1 by coIP assay using differentiated CAD cells and detected reciprocal coIP signals (Fig. 3e). More importantly, we observed that DISC1 overexpression enhances the interaction between KLF16 and SIN3A (Fig. 3f), indicating that DISC1 may act as a functional scaffold for SIN3A and KLF16. In the immunocytochemistry analyses using cultured mouse striatal neurons (DIV10), DISC1 and SIN3A were highly colocalized in the nucleus upon KLF16 co-expression (Fig. 3g–i). Taken together, these results demonstrate that DISC1 facilitates physical and functional interactions between KLF16 and SIN3A to direct the repressor activity toward the *D1r* locus.

### DISC1-Deficiency is Responsible for D1R Signaling and Related Behaviors

**Fig. 4:**
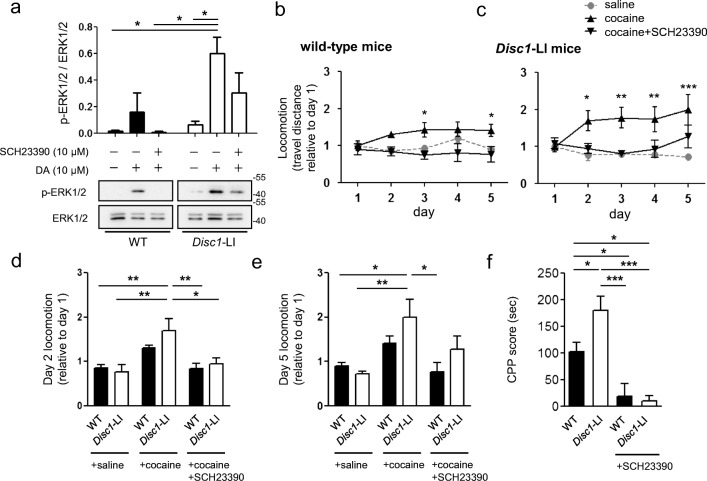
Effects of DISC1-deficiency on D1R signaling and related behaviors. **a** Enhanced phosphorylation of ERK1/2. Primary cultured mouse striatal neurons (DIV10) from *Disc1-*LI or wild-type mouse embryos (E15.5) were treated with DA (10 μM) for 30 min. SCH23390 (10 μM) was pretreated for 15 min. **b**, **c** Altered locomotor sensitization to cocaine in wild-type mice (**b**) and *Disc1*-LI mice (**c**). Mice were treated with saline or cocaine (20 mg/kg) in combination with SCH23390 (0.3 mg/kg) as indicated. Data represent total travel distances relative to that on day 1 (*n* = 6 for WT + cocaine and WT + cocaine + SCH23390; *n* = 7 for *Disc1*-LI + cocaine + SCH23390; *n* = 8 for the others). **d**, **e** Comparison of locomotion between wild-type mice and *Disc1*-LI mice on day 2 (**d**) and day 5 (**e**). **f** Cocaine CPP scores of wild-type (*n* = 11) and *Disc1*-LI mice (*n* = 8) and the effect of pretreatment of SCH23390 (*n* = 7 for each). Data represent mean ± SEM. *P < 0.05; **P < 0.01; ***P < 0.001; two-way ANOVA with post-hoc Bonferroni test (**b**, **c**); one-way ANOVA with post hoc Tukey test (**a**, **d**, **e, f**)

To examine whether the alteration in D1R protein expression under DISC1-deficiency is further linked to dopamine-mediated signaling, we analyzed the phosphorylation level of extracellular signal-regulated kinase 1/2 (ERK1/2), a D1R downstream signaling pathway [23], by western blotting. Striatal neurons cultured from *Disc1*-LI mouse embryos exhibited an enhanced p-ERK level in response to dopamine treatment. Pretreatment with SCH23390, a D1R antagonist, before dopamine, suppressed the enhanced phosphorylation of ERK1/2 (Fig. 4a). This result indicates that alterations in *D1r* transcription under DISC1-deficiency are transmitted to the downstream signaling pathway.

To test the behavioral effect of elevated D1R signaling by DISC1-deficiency, we performed cocaine-induced locomotor sensitization in combination with SCH23390 using *Disc1-*LI mice because D1R is known to be essential for cocaine-induced locomotor sensitization [24]. The doses of cocaine and SCH23390 were determined by preliminary experiments using wild-type mice (Supplementary Fig. 2). *Disc1*-LI mice showed more active locomotion than wild-type mice upon 20 mg/kg of cocaine, indicating exaggerated behavioral sensitization of *Disc1-*LI mice (Fig. 4b–e). In both wild-type and *Disc1*-LI mice, pretreatment with SCH23390 effectively blocked the development of locomotor sensitization to cocaine on days 2 and 5 (Fig. 4d, e), indicating that the exaggerated cocaine responses are due to alterations of D1R functionality. We also examined general motor function in the open field and rotarod tests. *Disc1*-LI mice did not show altered locomotive activity in the open field test and motor coordination in the rotarod test (Supplementary Fig. 3). These results indicate that presynaptic function of the striatum is not likely responsible for behavioral changes in locomotor sensitization. We next applied *Disc1*-LI mice to the conditioned place preference (CPP) to cocaine because D1R is involved in the acquisition of cocaine CPP [25]. DISC1 deficiency significantly increased cocaine CPP, and pretreatment with SCH23390 efficiently blocked the place preference (Fig. 4f), indicating that the elevated D1R level in *Disc1*-LI mice is contributing to the increased place preference. Taken together, the upregulation of D1R caused by DISC1-deficiency is responsible, at least in part, for behavioral changes in DISC1-deficient mice.

## Discussion

Here, we present a novel epigenetic regulatory mechanism for D1R expression that is achieved by the participation of DISC1, a factor associated with major mental illnesses. We demonstrated that DISC1 functions as a negative regulator of *D1r* transcription by physical interaction with KLF16 and recruitment of the SIN3A repressor complex to the *D1r* locus. Multiple studies have reported potential roles for DISC1 in the nucleus. Millar et al. [26] initially reported that the N-terminus of DISC1 (amino acid residues 1–358) is critical for its localization to the nucleus, and Sawamura et al. [19] narrowed down the region to amino acid residues 1–45. DISC1 is also reported to play a role in the transcriptional regulation of the *PDE4D9* gene in collaboration with ATF4 and N-CoR, which is under the control of D1R signaling [27]. As DISC1 loss-of-function results in a higher baseline level of PDE4D9, which is supposed to suppress D1R signaling, fine-tuning of D1R signaling via transcriptional feedback has been suggested. Our findings indicate that a parallel transcriptional mechanism exists in conjunction with DISC1 and KLF16. KLF16 induced nuclear localization of DISC1 and its recruitment to SIN3A complex, although it is not clear at this stage if the previously known nuclear localization signal (NLS) of DISC1 and KLF-induced accumulation of DISC1 in the nucleus are mechanistically interlinked. However, the coordinated recruitment of the two proteins into the nucleus is in good correlation with the concerted action of DISC1, KLF16, and SIN3A at the *D1r* locus, similar to that in DISC1/ATF4. These findings reveal a novel role of DISC1 as a general nuclear scaffold protein related to the transcriptional repression complex and suggest its functions for fine-tuning dopamine signaling, which is disrupted under DISC1 deficiency, thereby generating a hyper-dopaminoceptive condition. This finding is also in line with the evidence that dopaminergic pathways governed by dopamine receptors are altered in several neurological and psychiatric conditions such as schizophrenia [28]. Moreover, the enhanced level of dopamine in the nucleus accumbens was observed in drug addiction, obesity, or depression [29, 30]. In this regard, elevated D1R in the ventral striatum of *Disc1-*LI mice might be related to an aspect of these psychiatric disease pathologies.

We demonstrated that deficiency of DISC1 leads to the enhancement of cocaine-induced dopamine signaling through postsynaptic D1R. Previously, hyperactivity in response to psychostimulants has been shown in multiple DISC1 mutant and transgenic mouse models [11, 12]. The phenotype can be explained by the potential presynaptic functions of DISC1 in dopamine release or by its direct impact on postsynaptic dopamine signaling processes. The *Disc1-*LI mice used in this study did not show a difference in locomotive activity in the open field test and motor coordination in the rotarod test, implying that their presynaptic functionality related to dopamine release was not significantly affected. On the other hand, the augmented expression of *D1r* mRNA in the striatum of *Disc1-*LI is consistent with the findings in some other DISC1-deficient mice or dominant-negative DISC1 mice [11, 12]. Thus, the cocaine-induced hyperlocomotion in these mouse models can be easily related to the elevation of D1R in the striatal region, which is also supported by the reports that the D1R is essential for drug-induced locomotor sensitization [31]. The effects of DISC1-deficiency on the elevation of D1R and cocaine-induced behaviors might be in line with the comorbidity between substance abuse and other psychiatric disorders [32]. Of note, schizophrenia patients are more prone to abuse substances and increased psychotic effects from the substance [33–35]. Supporting this notion, phenotypes of DISC1 mutation or deficiency share some characteristics with addictive behaviors, and rare variants in DISC1 were associated with opioid dependence [36]. This phenomenon might be linked to the increased cocaine CPP of *Disc1*-LI mice shown in this study. Collectively, our results suggest that DISC1 is relevant to the interface among substance dependence and other psychiatric conditions.

### Experimental Procedures

#### Plasmid Constructs

Mouse *Klf16* cDNA was cloned in pEGFP-N1 (Clontech) and pcDNA 3.1/HA (Invitrogen) vectors. Mouse *Disc1* cDNA was cloned in pFlag-CMV2 (Sigma) vector, and mouse *Sin3a* was cloned in pcDNA 3.1/Myc-His (Invitrogen) vector.

#### Mice

Pregnant wild-type (B57BL/6) mice were obtained from Hyochang Science (Daegu, Republic of Korea) for striatal neuron culture. Production and management of wild-type (B57BL/6), *Disc1* (Δ2–3/Δ2–3) mutant (a kind gift from Dr. Kozo Kaibuchi, Nagoya University), and DISC1 locus impairment mouse (*Disc1-*LI, a kind gift from Dr. Akira Sawa, Johns Hopkins University School of Medicine) had been described previously [15, 37–39]. All mice were fed ad libitum in a 12 h light/12 h dark cycle for 12 weeks and subjected to behavior test or biochemical experiments. All animal procedures were approved by the Pohang University of Science and Technology Institutional Animal Care and Use Committee.

#### Primary Culture of Mouse Neurons

Primary culture of mouse embryonic striatal neuron was conducted as described previously [29, 40] with some modifications. Striatal regions were dissected from E15.5 mouse embryos in Hank’s balanced salt solution (HBSS, Invitrogen) without calcium or magnesium. The dissociated cells were diluted in the plating media (MEM (Invitrogen) supplemented with 1 M HEPES, pH 7.4, 10% glutamine, and 10% horse serum) and plated in 12-well or 24-well plates coated with poly-D-lysine and laminin. Three hours after the plating, the media were replaced with the culture media (MEM supplemented with 2% B27 (GIBCO), 10% glutamine, and 1% penicillin/streptomycin (GIBCO)).

#### Mammalian Cell Culture and Transfection

Neuroblastoma CAD cell line was maintained in DMEM (Hyclone) supplemented with 10% (*v*/*v*) fetal bovine serum (GIBCO) and 1% penicillin/streptomycin. The CAD cell line was a kind gift from Dr. MD Nguyen (University of Calgary). We confirmed its capacity of morphological differentiation upon serum deprivation. Cultured neurons and differentiated CAD cells were transfected using Lipofectamine 2000 (Invitrogen).

#### CoIP Assay

CoIP assay was performed on cell lysates in NP40 lysis buffer (150 mM NaCl, 50 mM Tris-HCl, pH 8.0, 1% NP40) supplemented with protease inhibitor (Pierce). Antibodies were added to the lysates and incubated for 4 h or overnight at 4 °C. Immunocomplexes were precipitated using protein A-agarose (Roche). The pellets were washed three times with the NP40 lysis buffer and prepared for subsequent SDS-PAGE and western blot assay.

#### Nuclear Fractionation

Cytoplasm- and nucleus-enriched lysates were prepared as described previously [41] with some modifications. Cultured cells were scraped in the lysis/extraction buffer containing 10 mM HEPES (pH 7.6), 0.5% NP40, 5% glycerol, 3 mM MgCl_2_, 40 mM KCl, 2 mM DTT, 0.5 mM PMSF, and protease inhibitor. After incubation on ice for 30 min, 10% of each lysate was kept as the whole lysate and the last was centrifuged at 1000*g*, 4 °C for 5 min. The supernatant was centrifuged at 13,000*g*, 4 °C for 10 min and kept as the enriched cytoplasm fraction. The pellet was washed twice with the lysis/extraction buffer and centrifugation at 1000*g*, then resuspended in the nuclear extraction buffer containing 10 mM HEPES (pH 7.9), 1.5 mM MgCl_2_, 420 mM NaCl, 0.1 mM EGTA, 25% glycerol, 0.5 mM DTT, 0.5 mM PMSF, and protease inhibitor. After incubation on ice for 30 min and sonication, cellular debris was removed by centrifugation at 13,000*g*, 4 °C for 10 min, and the supernatant was kept as the enriched nuclear fraction.

#### Antibodies and Western Blotting

Protein concentration was determined by the Bradford method. Fifteen to eighty micrograms of proteins was subjected to SDS-PAGE. The antibodies used are as follows: anti-DISC1 (ABN425, Millipore), anti-GFP (B-2, Santa Cruz Biotechnology), anti-Myc (9E10, sc-40, Santa Cruz Biotechnology), anti-GAPDH (6C5, Santa Cruz Biotechnology), anti-FLAG M2 (F1804, Sigma), anti-FLAG (PA1-984B, ABR), anti-HA (A190-108A, Bethyl), anti-Lamin B (B-10, Santa Cruz Biotechnology), anti-Actin (I-19, Santa Cruz Biotechnology), anti-SIN3A (AK-11, Santa Cruz Biotechnology), anti-D1R (SG2-D1a, Santa Cruz Biotechnology), p44/42 MAPK (ERK 1/2) (1:1000; #9102, cell signaling), P-p44/42 MAPK (phospho-ERK 1/2) (1:1000; #4377, cell signaling), rabbit IgG (ab37415, Abcam), and goat anti-mouse and goat anti-rabbit HRP-labeled secondary antibodies (1:15,000; Thermo Fisher Scientific and Promega, respectively). For D1R western blot, the protein samples were denatured at 37 °C for an hour. For phospho-ERK 1/2 and ERK 1/2 western blot, cells were treated with 10 μM DA (H8502, Sigma) for 30 min in the 37 °C incubator (5% CO_2_) before lysis. Ten microgram SCH23390 (D054, Sigma) was pretreated 15 min before DA treatment.

#### Immunofluorescence Microscopy

For immunofluorescence imaging, cells were fixed in 4% paraformaldehyde dissolved in PBS for 10 min and blocked with PBS containing 2% goat serum and 0.2% Triton X-100. Cells were incubated with primary antibodies for 2–4 h and visualized with secondary antibodies conjugated with Alexa Fluor 568, and Fluor Alexa 647 (Invitrogen), respectively. Hoechst 33342 dye (Thermo Scientific) was used for nuclear staining. Images were obtained by a laser scanning confocal microscopy (FV3000, Olympus) and Mander’s overlap coefficients and Pearson’s correlation coefficients were analyzed by CellSens software (Olympus). All images were processed with the auto-threshold option of CellSens software, and none of the images were manipulated manually.

#### Chromatin Immunoprecipitation (ChIP) Assay

ChIP assays were carried out from differentiated CAD cells as described previously [42]. Polyclonal rabbit anti-acetylated H3 K9 and K14; anti-acetylated H4 K5, K8, K12, and K16 (#06–599 and #06–866, respectively Millipore); anti-GFP (B-2, Santa Cruz Biotechnology); and anti-Myc (9E10, Santa Cruz Biotechnology) antibodies were used for ChIP. The primers correspond to the sequences at − 1614 to − 1308 base pairs relative to the translation start site of the mouse *D1r* gene.

- D1R F: 5′-GGTGGAGGAGGACTGGTGTCAA-3′
- D1R R: 5′-CTTGGAAATCACTTTGCCTGGA-3′
- GAPDH F: 5′-AACGACCCCTTCATTGAC-3′
- GAPDH R: 5′-TCCACGACATACGCAC-3′

#### Real-time Quantitative RT-PCR

Total RNA was extracted using Tri-Solution (Bioscience Technology) from striatal tissues or differentiated CAD cells. For reverse transcription, 1 μg of RNA was used to synthesize first-strand cDNA using the ImProm-II™ Reverse Transcription System (Promega). For real-time qPCR, we used FastStart Universal SYBR Green Master (Rox) (Roche) and the StepOnePlus real-time PCR system (Applied Biosystems) according to the manufacturer’s protocol using the following primers:

- D1R F: 5′-GGGCCCTACTACGAATAATG-3′
- D1R R: 5′-CATAGTCCAATATGACCGATAAG-3′
- D2R F: 5′-GCTCAGGAGCTGGAAATGGAGAT-3′
- D2R R: 5′-CTTCCTGCGGCTCATCGTCTT-3′,
- D3R F: 5′-GTCCTGCCCTCTCCTCTTTGGTTT-3′
- D3R R: 5′-AGTCTACGGTGCCCTGTTTAC-3′
- D4R F: 5′-TGCCCTCAACCCCATCATCTACAC-3′
- D4R R: 5′-AATACTTCCGACCCCCAACCCT-3′
- D5R F: 5′-GGGAGATCGCTGCTGCCTATGTC-3′
- D5R R: 5′-TTTTAGAGTGGTGAGTGGGGGTTA-3′
- GAPDH F: 5′-CACTGAAGGGCATCTTGG-3′
- GAPDH R: 5′-TTACTCCTTGGAGGCCATG-3′

#### Cocaine Sensitization

Cocaine sensitization was conducted as described previously [43]. All mice were allowed to rest in the testing room for 30 min prior to performing any behavioral assessment in the open field test during the cocaine sensitization paradigm. Mice were administered with saline (0.9% NaCl), cocaine (20 mg/kg, Tocris, UK), or SCH23390 (0.3 mg/kg) by an intraperitoneal injection (4 mL/kg body weight) for 5 days. SCH23390 was treated 15 min before measurement of locomotor activity. Cocaine was given just before the assay.

#### Open Field Test

All mice were allowed to rest in the testing room for 30 min prior to performing any behavioral assessment in the open field test. Animals were placed in the center of the arena (40 × 60 × 20 cm, white background) and their behaviors were recorded for 30 min by a video tracking system (Smart v2.5, Panlab).

#### Rotarod Test

Mice were trained in an elevated rotating rod (Panlab, Harvard Apparatus) at a fixed speed of 10 rpm, and then tested in an accelerating rod from 4 to 40 rpm for 2 min, where the latency to fall in seconds was registered.

#### Conditioned Place Preference (CPP) Test

Mice were conditioned to cocaine using a conditioning apparatus, which consisted of three distinct environment chambers (white side, gray middle, and black side). On day 1, mice were placed in the middle chamber and allowed to explore the conditioning apparatus. On days 2 and 3, mice received two pairings per day: saline (0.9%, 1 mL/kg; i.p.) in the morning and cocaine (20 mg/kg; i.p.) in the evening on the opposite side of the place preference chambers. SCH23390 (0.3 mg/kg; i.p.) was injected 15 min ahead of the cocaine administration. At the post-test on day 4, mice were placed again in the middle chamber with free access to all chambers, and the time spent in each side was quantified. Data represents the time spent on the cocaine-paired side minus the time spent on the saline-paired side (CPP score).

### Statistical Analysis

All data are presented as mean ± SEM. Statistical significances were analyzed using one-way ANOVA with post-hoc Tukey tests, two-way ANOVA with post-hoc Bonferroni tests, or two-tailed independent-samples Student’s *t* tests using the GraphPad Prism software for Windows (version 5). Statistical significance is indicated as follows: *P < 0.05; **P < 0.01; ***P < 0.001.

## Supplementary Data

ESM 1 (DOCX 216 kb)
