# Supplementary material for: Dopamine D1 Receptor (D1R) Expression Is Controlled by a Transcriptional Repressor Complex Containing DISC1
Source: Mol Neurobiol. 2019 Mar 26;56(10):6725–35. doi: 10.1007/s12035-019-1566-6 (PMC6728282; doi:10.1007/s12035-019-1566-6)

Molecular Neurobiology

Dopamine D1 Receptor (D1R) Expression is Controlled by a Transcriptional Repressor Complex Containing DISC1

**Yeongjun Suh^1#^, Su-Jin Noh^1#^, Saebom Lee^2,3#^, Bo Kyoung Suh^1^, Su Been Lee^1^, Jinhyuk Choi^1^, Jaehoon Jeong^4^, Sangjune Kim^5,6^, Sang Ki Park^1^***

^1^Department of Life Sciences, Pohang University of Science and Technology, 37673, Pohang, Republic of Korea

Current affiliation: ^2^The Russell H. Morgan Department of Radiology and Radiological Sciences, The Johns Hopkins University of School of Medicine, Baltimore, MD, USA, ^3^The Center for Nanomedicine at Wilmer Eye Institute, The Johns Hopkins University of School of Medicine, Baltimore, MD, USA ^4^National Institute of Neurological Disorders and Stroke, National Institutes of Health, Bethesda, MD, USA, ^5^Neurodegeneration and Stem Cell Programs, Institute for Cell Engineering, The Johns Hopkins University of School of Medicine, Baltimore, MD, USA ^6^Department of Neurology, The Johns Hopkins University School of Medicine, Baltimore, MD, USA

^#^These authors contributed equally to this work.

*To whom correspondence should be addressed: Sang Ki Park: Department of Life Sciences, Pohang University of Science and Technology, 37673, Pohang, Republic of Korea; [skpark@postech.ac.kr](mailto:skpark@postech.ac.kr); (+82) 54-279-2349

**Supplementary Material**

**Supplementary Figure Captions**

**Supplementary Fig. 1** Effect of DISC1 on the expression of dopamine receptor genes. **a, b** Relative transcript abundances of DRs in the striatum of adult WT and *Disc1*-LI mice, which are normalized to D1R of WT mice (**a)**; normalized to WT, (n=5 for D3R, n=6 for the others). **P*<0.05; two-tailed t-test

**Supplementary Fig. 2** Cocaine-induced locomotor sensitization for dose determination of cocaine (**a**), and SCH23390 (**b**) in WT mice (n=3). **P*<0.05; ***P*<0.01; ****P*<0.001; two-way ANOVA with *post hoc* Bonferroni test, compared to the saline group

**Supplementary Fig. 3** General motor function of *Disc1*-LI mice. **a** Open field test. Data represent total travel distance (n=5; two-tailed t-test) **b** Rotarod test. Data represent latency time and rpm to fall (n=15 for WT; n=9 for *Disc1*-LI; ns=not significant; two-tailed t-test)

Supplementary Fig.1


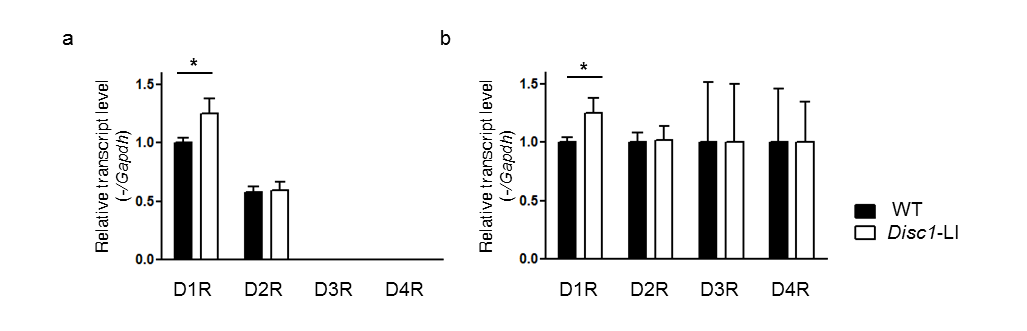


Supplementary Fig.2


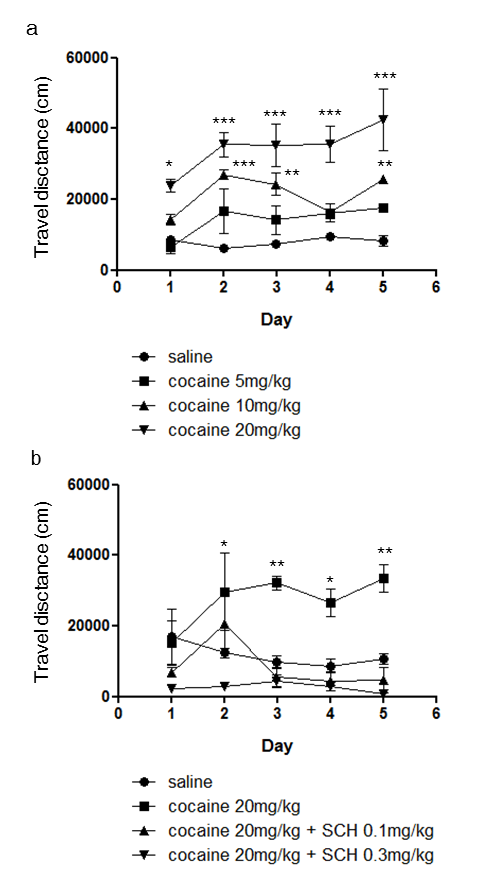


Supplementary Fig.3


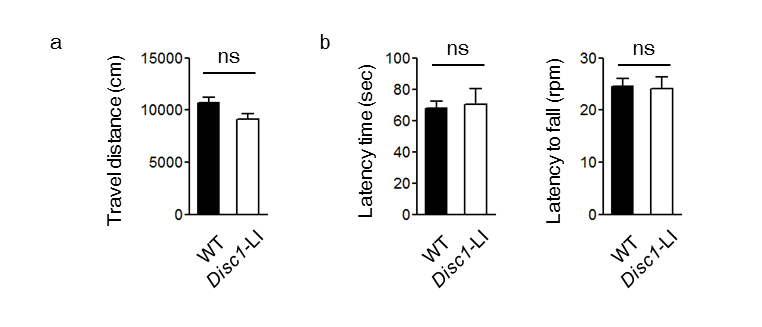

Supplement: ESM 1 — (DOCX 216 kb) [file 12035_2019_1566_MOESM1_ESM.docx]
